# Tumor-Infiltrating Lymphocytes in Localized Prostate Cancer: Do They Play an Important Role?

**DOI:** 10.7759/cureus.34007

**Published:** 2023-01-20

**Authors:** Marta Vilaça, João Correia Pinto, Helena Magalhães, Frederico Reis, Alexandra Mesquita

**Affiliations:** 1 Oncology Department, Hospital Pedro Hispano, Matosinhos, PRT; 2 Pathology Department, Hospital Pedro Hispano, Matosinhos, PRT; 3 Urology Department, Hospital Pedro Hispano, Matosinhos, PRT

**Keywords:** tumor-infiltrating lymphocytes, pathology, immunotherapy, biochemical recurrence, prostate cancer

## Abstract

Background

Localized prostate cancer is a heterogeneous entity, and new biomarkers are required for risk stratification. This study aimed to characterize tumor-infiltrating lymphocytes (TILs) in localized prostate cancer and assess their potential prognostic markers.

Methodology

Radical prostatectomy specimens were analyzed to determine infiltration levels of CD4+, CD8+, T cells, and B cells (characterized by CD20+ cells) in the tumor tissue using immunohistochemistry and the recommendations of the International TILs Working Group 2014. The clinical endpoint was biochemical recurrence (BCR), and the study sample was divided into two cohorts (cohort 1: without BCR; cohort 2: with BCR). Prognostic markers were assessed using Kaplan-Meier and univariate/multivariate Cox regression analysis using SPSS version 25 (IBM Corp., Armonk, NY, USA).

Results

We included 96 patients in this study. BCR occurred in 51% of the patients. Normal TILs infiltration was found in most of the patients (41/31, 87%/63%). T CD4+ infiltration was statistically superior in cohort 2. This enrichment was associated with BCR (p < 0.05; log-rank test). After adjustment for routine clinical variables and Gleason grade groups (grade group ≤2 and grade group ≥3), it remained an independent prognostic variable of early BCR (p < 0.05; multivariate Cox regression).

Conclusions

This study showed that immune cell infiltration appears to be an important prognostic variable for early recurrence in localized prostate cancer.

## Introduction

Prostate cancer (PC) is the second most frequent cancer worldwide. It was the fifth leading cause of cancer death among men in 2020 [1]. In localized disease, patients can be treated with surgery or radiotherapy based on stage, Gleason score (GS), and serum prostate-specific antigen (PSA) [2]. Unfortunately, in about 30% of patients, biochemical recurrence (BCR) after local treatment occurs, with half of the patients progressing to metastatic disease [3-5]. In addition to prostate-specific antigen doubling time (PSADT), which has been shown to be a predictor of PC progression, metastases, and PC-specific mortality (PCSM), novel prognostic markers are needed to improve risk stratification and guide more personalized treatment decisions [6]. At the same time, because there are limited treatment options for metastatic and treatment-resistant diseases, new treatment strategies are needed [7].

Recent studies have shown that tumor-infiltrating lymphocytes (TILs) and their microenvironment play an important role in cancer progression and metastasis. It was demonstrated that a strong infiltration of immune cells, especially T CD8+, was associated with a positive clinical outcome in melanoma, head and neck, breast, ovarian, colorectal, and lung cancer [8]. Moreover, in the era of immunotherapy, TILs are speculated to be a possible predictive marker of treatment efficacy [9,10]. However, the role of TILs in PC as well as their composition remains controversial [11]. The prostate tumor microenvironment is considered immunologically “cold” with fewer infiltration of immune cells when compared to “hot” cancer, such as melanoma, lung, or bladder cancer [12]. Even so, the prostate tumor microenvironment is not devoid of immune cell infiltrates, and the majority of studies have shown predominant infiltration by CD4+ T lymphocytes and only a limited influx of CD8+ T cells and B cells [13-17]. An association between the tumor-infiltrating regulatory T cells and adverse outcomes after radical prostatectomy (RP) has been reported, with shorter BCR-free survival and increased risk of metastasis [13,15,17]. The role of cytotoxic T cells as a prognostic biomarker remains uncertain, with some studies showing adverse outcomes and others reporting improved survival after RP with high CD8+ T cells [11,13,18]. Hence, the biological constitution of TILs, as well as their implication in patient outcomes, needs further investigation.

This study aimed to characterize TILs in patients who underwent RP due to localized prostate cancer and assess their importance as a prognostic biomarker in early recurrence.

## Materials and methods

**Figure 1:**
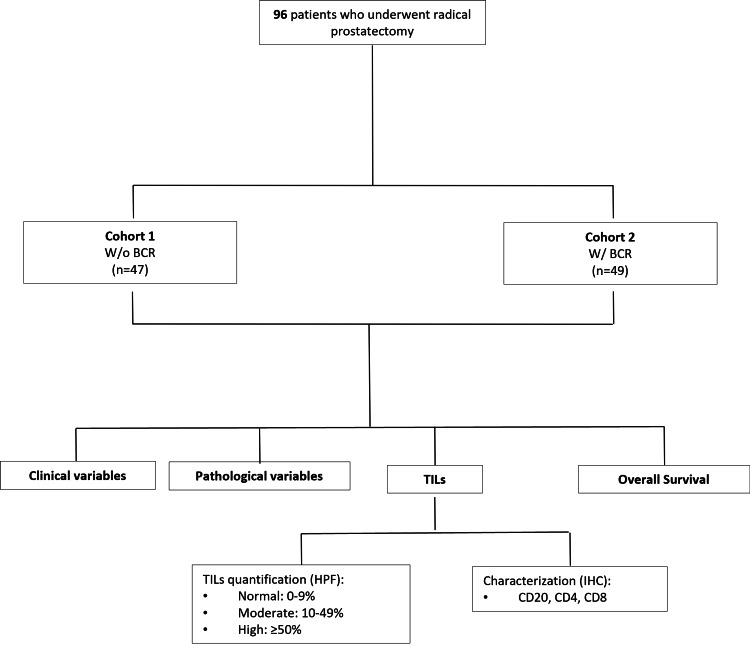
Flowchart of the study. Gleason grade group (grade group ≤2 and ≥ 3), cribriform pattern, and pathological stage were included as pathological variables BCR = biochemical recurrence; TILs = tumor-infiltrating lymphocytes; HPF = high-power field; IHC = immunohistochemistry

The project was approved by the Ethics Committee of Pedro Hispano Hospital, Matosinhos, Portugal (reference number: 65/CES/JAS). Due to its retrospective nature, without patients’ direct involvement and no risks for them, as well as the potential benefit of this work, the investigators requested an informed consent waiver which was approved by the local ethics committee. The flowchart of the study is shown in Figure 1.

Study patients

A retrospective cohort of 96 patients who underwent RP at a secondary care hospital with 354 beds, between January 2013 and December 2015, was studied. Clinical variables and outcomes were collected from the patient database. BCR was defined as the time from RP until PSA increased. It was determined as undetectable PSA after RP with subsequent increase to ≥0.2 ng/mL on two or more consecutive determinations. Patients with persistent elevation of PSA after RP were excluded. According to the presence or absence of BCR, the sample was divided into two cohorts. Patients without BCR were included in cohort 1 and those with BCR were included in cohort 2. The primary clinical outcome was BCR. Overall survival (OS) was defined as the time from RP until death due to any cause or last follow-up. For this study, all RP specimens were regraded according to the 2014 International Society of Urological Pathology Gleason grading by a trained pathologist.

Evaluation of tumor-infiltrating lymphocytes and immunohistochemistry

All blocks from RP were evaluated by a trained pathologist. TIL evaluation was made according to the International TILs Working Group 2014 methodology validated in breast cancer in the borders of invasive PC [19]. TIL quantification was categorized, based on the area occupied by mononuclear inflammatory cells over the total intratumoral stromal area, as normal if they were present in 0-9% per high-powered field, moderate in 10-49%, and high in ≥50%. Using immunohistochemistry, we characterized the TIL sub-population, identifying the expression of CD20, CD4, and CD8 receptors. A semi-quantitative evaluation was made, with a ratio between the immune cells and all TILs present in the high-powered field.

Statistical analysis

Statistical analysis was performed using SPSS version 25 (IBM Corp., Armonk, NY, USA). Patients’ characteristics were reported using the median and interquartile range for continuous variables and relative frequencies for categorical variables. Comparisons were made between the two cohorts (without BCR vs. with BCR) using the Mann-Whitney and chi-square tests for continuous and categorical variables, respectively. Correlation between TIL sub-population was made using the Spearman correlation rank test. The time to BCR and OS was summarized using standard Kaplan-Meier methods, with estimates of the median at 95% confidence intervals (CIs). Comparisons were made using the log-rank test. Cox regression models were used to evaluate the association between clinical and pathological variables and TIL subtypes and time to BCR. All analyses were completed with a significance level of 0.05.

## Results

Patient demographic and clinicopathological characteristics

Between January 2013 and December 2015, 96 patients underwent RP due to prostate adenocarcinoma. Of this, 49 (51%) patients had BCR during the follow-up, and the median time to this occurrence was 33 months (range = 4-95 months). In total, 23 (47%) patients had early BCR (i.e., BCR in the first 33 months). Patient demographic, clinical, and histopathological characteristics by BCR are presented in Table 1. In this study, higher PSA levels before RP, higher GS, the presence of cribriform glands, and higher TIL scores were statistically more frequent in cohort 2. The follow-up time was also longer in cohort 2 (88 months in cohort 2 versus 80 months in cohort 1; p = 0.003).

**Table 1:** Patient characteristics. BCR = biochemical recurrence; ECOG = Eastern Cooperative Oncology Group; IQR = interquartile range; n = number; PSA = prostate-specific antigen; RP = radical prostatectomy; T = tumor; TILs = tumor-infiltrating lymphocytes

Variables	Cohort 1 (no BCR) (n = 47)	Cohort 2 (BCR) (n = 49)	P-value (α = 0.05)
Age at RP (year), median (IQR)	62 (60-63)	66 (52-72)	0.607
ECOG at RP, n (%)			0.265
0	16 (34%)	21 (43%)	
1	29 (62%)	28 (57%)	
2	2 (4%)	0 (0%)	
Comorbidities, n (%)			
Hypertension	23 (49%)	23 (47%)	0.838
Diabetes	9 (19%)	8 (16%)	0.793
Smoker	12 (26%)	14 (28%)	0.921
Body mass index (kg/m^2^), n (%)			0.277
<18.5	0 (0%)	1 (2%)	
18.5–24.9	13 (28%)	15 (31%)	
25–29.9	24 (51%)	24 (49%)	
30–34.9	10 (21%)	6 (12%)	
≥40	0 (0%)	3 (6%)	
Pre-RP PSA, ng/mL, median (IQR)	7 (6.5-7.7)	11 (10.4-15)	<0.001
RP Gleason score, n (%)			<0.001
6	13 (28%)	5 (10%)	
7 (3+4)	30 (64%)	14 (29%)	
7 (4+3)	4 (9%)	24 (49%)	
>7	0 (0%)	6 (12%)	
Pathological T-stage, n (%)			0.74
≤pT2	35 (74%)	28 (57%)	
≥pT3	12 (26%)	21 (43%)	
Presence of cribriform pattern, n (%)	6 (13%)	29 (59%)	<0.001
Surgical margins, n (%)			0.077
Negative	39 (83%)	33 (67%)	
Positive	8 (17%)	16 (33%)	
Adjuvant radiotherapy, n (%)	4 (9%)	10 (20%)	0.038
TILs score, n (%)			0.007
0–9%	41 (87%)	31 (63%)	
10–49%	6 (13%)	18 (37%)	
≥50%	0 (0%)	0 (0%)	
Follow-up (months), median (IQR)	80 (70-83)	88 (85-92)	0.003
Died during the follow-up, n (%)	4 (9%)	3 (6%)	0.527

Only three (7%) patients in cohort 2 developed metastatic disease during the follow-up. The median time between BCR and metastatic disease was 33 months (range = 19-48 months). The median disease-free survival was 81 months (95% CI = 74.4-87.5). The median OS was not reached at the time of follow-up. Only two patients in cohort 2 died from PC, while the others died from other causes.

Evaluation of tumor-infiltrating lymphocyte density and sub-classification by immunohistochemistry

To investigate the distribution and level of TILs in localized PC, we performed immunohistochemistry to CD20, CD4, and CD8 in intratumoral tissue. T-helper cells (CD4+) represented the most frequent sub-population of lymphocytes present in more than 60% of the sample. Table 2 shows the results of the TIL analysis in this population. A negative correlation between T CD4+ lymphocytes and T CD8+ (p < 0.001) was found in both groups on the Spearman correlation.

**Table 2:** TIL characteristics. n = number; BCR = biochemical recurrence; TILs = tumor-infiltrating lymphocytes

TILs sub-classification	Cohort 1 (no BCR) (n = 47)	Cohort 2 (BCR) (n = 49)	P-value (α = 0.05)
B cells (CD20+) % TILs			0.911
<5%	8 (17%)	9 (18%)	
5–25%	34 (72%)	36 (73%)	
26–75%	5 (11%)	4 (8%)	
>75%	0 (0%)	0 (0%)	
T CD4+ % TILs			<0.001
<5%	1 (2%)	0 (0%)	
5–25%	29 (62%)	0 (0%)	
26–75%	7 (15%)	5 (10%)	
>75%	10 (21%)	44 (90%)	
T CD8+ % TILs			<0.001
<5% of TILs	0 (0%)	4 (8%)	
5–25%	10 (21%)	40 (82%)	
26–75%	7 (15%)	5 (10%)	
>75%	30 (64%)	0 (0%)	

Predictors of biochemical recurrence

In this study, high levels of infiltrating T CD4+ lymphocytes were associated with BCR on the Kaplan-Meier assessment (p < 0.001, log-rank test). On Cox regression univariate analysis, high levels of T CD4+, lower levels of T CD8+, high preoperative PSA, high GS, the presence of cribriform glands, and higher levels of TILs were predictors of BCR (Table 3).

**Table 3:** Univariate and multivariate Cox regression of BCR. BCR = biochemical recurrence; HR = hazard ratio; PSA = prostate-specific antigen; RP = radical prostatectomy; T = tumor; TILs = tumor-infiltrating lymphocytes

Univariate analysis			Multivariate analysis	
Variables	HR (95% CI)	P-value	HR (95% CI)	P-value
TILs				
T CD4+	5.8 (2.74-12.19)	<0.001	5.53 (1.17-27.39)	0.036
T CD8+	0.33 (0.22-0.48)	<0.001	1.1 (0.31-3.95)	0.882
B Cells (CD20+)	0.9 (0.53-1.49)	0.651		
Pre-RP PSA	1.07 (1.04-1.10)	<0.001	1.05 (1.01-1.09)	0.011
RP Gleason score	2.5 (1.8-3.6)	<0.001	1.6 (1.01-2.5)	0.044
Presence of cribriform pattern	3.5 (1.9-6.2)	<0.001	2.2 (1.2-4.3)	0.016
TIL score	2.6 (1.43-4.67)	0.002	1.6 (0.85-2.98)	0.147

After adjusting for routine clinical variables in multivariate Cox regression analysis, a high level of infiltrating T CD4+ cells remained independent predictors of BCR (hazards ratio = 5.53, 95% CI = 1.17-27.39; p = 0.036).

## Discussion

In this retrospective study, we characterized the immune cell composition of TILs in early PC using immunohistochemistry and correlated them with BCR. We found that, besides being a tumor with limited lymphocyte infiltration, there was a selective enrichment for CD4+ T lymphocytes, while CD8+ T cells and B cells were selectively depleted, indicating a complex biological role for infiltrating immune cells in PC development and/or progression. Furthermore, we found that higher levels of T CD4+ lymphocyte infiltration were significant adverse predictors of postoperative BCR. These results can be used to improve risk stratification groups in localized PC and guide more personalized treatment decisions, for instance, by identifying patients who have a higher risk of disease recurrence and can benefit from treatment intensification. At the same time, it can identify an important therapeutic target that can be explored for the treatment of metastatic disease.

In this study, we found that more than 50% of patients had BCR which is higher than what is described in the literature [3,4]. We cannot fully account for this clinical outcome in our study cohort; it may be due to late presentation, or some unknown characteristics of our patient population such as enrichment for Gleason groups 3, 4, and 5. Nevertheless, in the study by Andersen et al., in cohort 1, almost 50% of the patients had BCR, which included 470 patients [17]. Hence, the real BCR is not yet well established.

Overall, we found that PC is not an immune cell-enriched tumor, with only limited TIL infiltration, which follows what has been described in the literature [13]. In addition to that, we found selective enrichment in the prostate cancer epithelium of T CD4+ cells with sparse infiltration of T CD8+ and B cells. This corroborates five earlier studies that reported lower cytotoxic lymphocyte levels in PC epithelium and CD4+ T lymphocyte enrichment in the prostate [12,17,19-21]. In contrast to our study, Anderson et al. have shown significant enrichment in B cells and T regulator (Treg) cells, with no significant change in T-helper (Th) cell levels. In this study, Th cell was defined as CD3+CD8-FoxP3- cells and Treg as CD3+CD8-FoxP3+, which can explain the different classifications of T cells. At the same time, they compared B cells in benign versus malignant prostate tissue which could explain the B-cell enrichment in malignant tissue [17]. Because we only evaluated malignant tissue, we could not make this comparison.

Our results identified and validated infiltrating T CD4+ as an adverse prognosis factor for PC patient outcomes, with an increased risk of BCR. This result is in accordance with previous studies that reported an association between high levels of infiltrating Tregs and poor prognosis of PC [12,17,22-24]. It has been reported that Tregs exert an immune-suppressive environment, which can promote tumor development and progression in PC. Thus, the manipulation of Treg cells is a promising anticancer therapeutic strategy [25]. Unfortunately, in our study, we did not categorize the CD4+ population into Th or Tregs so we cannot make this assumption.

It has been demonstrated that, in colon cancer, an internationally validated immune score is predictive of time to disease recurrence independent of existing prognostic factors, such as age, sex, tumor stage, node stage, and MSI. Of all clinical parameters, the immune score had the highest relative contribution to the risk of recurrence [9]. In this study, we showed that higher TIL infiltration was associated with a worse prognosis in patients with localized PC.

TIL cells, in particular T cells, represent the cellular support to immunotherapy treatment in cancer, and a better understanding of immune cells in the tumor microenvironmental is essential for the development of novel therapeutic targets, as well as new biomarkers of prognosis and response to treatments. Therefore, a systematic overview of the characteristics of TILs in different cancers is needed to elucidate the differences in immune response among different cancer types [26]. The so-called “cold” tumors are distinguished by a lack of infiltration for T cells, thus representing low responses to the programmed cell death-1/Programmed cell death ligand-1 blockade. An important investigation line is a chance of transforming a tumor from a non-inflamed into an inflamed immune contexture by combining therapeutic options, which will render it more sensitive to immunotherapy [27]. We saw a lack of TIL infiltration, with the most amount of CD4+ T cells in patients with localized PC. Therefore, an important investigation line in PC will be a combination of immunotherapy with other therapeutic options, such as chemotherapy and radiotherapy, to convert this “cold” tumor into immunogenic cancer.

The major strengths of our study were (1) the use of a validated methodology to evaluate TILs in PC, (2) a systematic method for characterization of immune cell landscape in the PC microenvironment, and (3) establishing a correlation between TILs and clinical and biomarkers used in clinical practice.

However, our study has some limitations. First, we used paraffin-fixed samples, which can modify the tumor microenvironment through the fixation process. Second, we only analyzed TILs, and it is important to use a control group with benign pathology to compare. Third, we only identified CD8+ and CD4+ T cells. It was important to correlate this with CD3+ and FoxP3+ so we could differentiate TILs in Th, Tregs, and T cytotoxic cells. Fourth, the assessment was semi-quantitative with manual counts of lymphocytes versus glandular prostate epithelium. more detailed analyses such as artificial intelligence-aided image analysis with whole-slide images can be performed in the future. Finally, we used BCR as the primary endpoint for prognostic evaluation due to the limited follow-up time, which is only a surrogate for PC aggressiveness. Hence, future studies of TILs as a prognostic biomarker in the metastatic setting as well as the assessment of treatment response correlated with TIL infiltration should be conducted.

## Conclusions

We demonstrate that besides the prostate being a “cold” tumor, immune cell characterization is important as a prognostic biomarker for BCR. Our results may be used to improve risk stratification for localized PC as well as patient selection for immune therapeutic approaches, with new treatment strategies.
